# A Dead Tooth

**Published:** 1885-03

**Authors:** 


### A DEAD TOOTH.

A case has just come under my notice which is interesting, in as far as it illustrates how long a tooth may remain in the jaw after the death of its pulp, without causing trouble. The patient, a lady aged 27, tells me that when a child of about eight years old she was one day playing in a crib, and falling against the side of it, struck her two upper central incisors so violently as to break off the tip of one of them. This broken tooth had given her no trouble, nor had its fellow until about a week ago, when the gum around the latter one became swollen, and the tooth loose and painful. This morning I examined the two teeth and found them both free from caries. No gumboil was present, nor had there ever been one, and the swelling of the gum had quite subsided.

The broken tooth was of the normal color, but the other, which had been loose and painful, was of a darker shade.

All the evidence tended to show that this tooth was dead. accordingly I drilled through the lingual slope of the enamel into the pulp cavity. There I found only a little pus and no vestige of the pulp.

After due treatment I propose to fill the tooth to the end of the root canal and expect it will continue to be of service. It seems to be fair to conclude that the pulp of this tooth was killed at the time of the accident in childhood, in which case it had remained without causing trouble for a period of nineteen years.—*Dental Record.*

